# Exploiting the brain's network structure in identifying ADHD subjects

**DOI:** 10.3389/fnsys.2012.00075

**Published:** 2012-11-16

**Authors:** Soumyabrata Dey, A. Ravishankar Rao, Mubarak Shah

**Affiliations:** ^1^Computer Vision Lab, Department of Electrical Engineering and Computer Science, University of Central FloridaOrlando, FL, USA; ^2^IBM T.J. Watson Research CenterYorktown Heights, NY, USA

**Keywords:** attention deficit hyperactive disorder, default mode network, functional magnetic resonance image, linear discriminant analysis, principal component analysis

## Abstract

Attention Deficit Hyperactive Disorder (ADHD) is a common behavioral problem affecting children. In this work, we investigate the automatic classification of ADHD subjects using the resting state functional magnetic resonance imaging (fMRI) sequences of the brain. We show that brain can be modeled as a functional network, and certain properties of the networks differ in ADHD subjects from control subjects. We compute the pairwise correlation of brain voxels' activity over the time frame of the experimental protocol which helps to model the function of a brain as a network. Different network features are computed for each of the voxels constructing the network. The concatenation of the network features of all the voxels in a brain serves as the feature vector. Feature vectors from a set of subjects are then used to train a PCA-LDA (principal component analysis-linear discriminant analysis) based classifier. We hypothesized that ADHD related differences lie in some specific regions of brain and using features only from those regions are sufficient to discriminate ADHD and control subjects. We propose a method to create a brain mask which includes the useful regions only and demonstrate that using the feature from the masked regions improves classification accuracy on the test data set. We train our classifier with 776 subjects, and test on 171 subjects provided by the Neuro Bureau for the ADHD-200 challenge. We demonstrate the utility of graph-motif features, specifically the maps that represent the frequency of participation of voxels in network cycles of length 3. The best classification performance (69.59%) is achieved using 3-cycle map features with masking. Our proposed approach holds promise in being able to diagnose and understand the disorder.

## 1. Introduction

Attention Deficit Hyperactivity Disorder (ADHD) is a common behavioral disorder affecting children. Approximately 3–5% of school aged children are diagnosed with ADHD. Currently, no well-known biological measure exists to diagnose ADHD. Instead doctors rely on behavioral symptoms to identify it. To understand the cause of the disorder more fundamentally, researchers are using new structural and functional imaging tools like MRI and functional magnetic resonance imaging (fMRI). fMRI has been widely used to study the functioning of brain. It provides high quality visualization of spatio-temporal activity within a brain, which can be used to compare the functioning of normal brains against those with disorders.

fMRI has been used for different functional studies of brain. Some of the researchers have used task-related fMRI data, in which the test subjects perform conscious tasks depending on the input stimuli. Others used resting state brain fMRI data. The brain remains active even during rest, when it is not engaged in an attentive task. Raichle et al. (2001) identified several brain areas such as the medial prefrontal cortex (MPFC), posterior cingulate cortex (PCC), and precuneus that are active during rest. These areas form part of a functional network known as the resting-state network or default mode network (DMN) (Greicius et al., 2004; Damoiseaux et al., 2006). The literature (Greicius et al., 2004; Cherkassky et al., 2006; Damoiseaux et al., 2006) tends to use interchangeably the concepts of resting state brain networks and the DMN as defined by Raichle et al. (2001). We compare the brain regions that we have found in the current ADHD data set with the components of the DMN described by Raichle et al. (2001). It is believed that the DMN may be responsible for synchronizing all parts of the brain's activity; disruptions to this network may cause a number of complex brain disorders (Raichle, 2010). Researchers have studied neural substrates relevant to ADHD related behaviors, such as attention lapses, and identified the DMN as the key areas to better understand the problem (Weissman et al., 2006). In this study we use the resting state brain fMRI data and hypothesize that the differences between ADHD conditioned and control brains lie in the variation of functional connections of DMN.

Many studies have been performed to identify functional differences related to ADHD. Most of the approaches use group label analysis to deduce the statistical differences between ADHD conditioned and control groups. Structural MRI analysis suggests that there are abnormalities in ADHD brains, specifically in the frontal lobes, basal ganglia, parietal lobe, occipital lobe, and cerebellum (Castellanos et al., 1996; Overmeyer et al., 2001; Sowell et al., 2003; Seidman et al., 2006). In another set of studies, ADHD brains were analyzed using task-related fMRI data. Bush et al. (1999) found significant low activity in the anterior cingulate cortex when ADHD subjects were asked to perform the Counting Stroop during fMRI. Durston (2003) showed that ADHD conditioned children have difficulty performing the go/nogo task and display decreased activity in the frontostriatal regions. Teicher et al. (2000) demonstrated that boys with ADHD have higher T2 relaxation time in the putamen which is directly connected to a child's capacity to sit still. A third set of work was done using the resting state brain fMRI to locate any abnormalities in the DMN. Castellanos et al. (2008) performed Generalized Linear Model based regression analysis on the whole brain with respect to three frontal foci of DMN, and found low negative correlated activity in precuneus/anterior cingulate cortex in ADHD subjects. Tian et al. (2006) found functional abnormalities in the dorsal anterior cingulate cortex; Cao et al. (2006) showed decreased regional homogeneity in the frontal-striatal-cerebellar circuits, but increased regional homogeneity in the occipital cortex among boys with ADHD. Zang et al. (2007) verified decreased amplitude of low-frequency fluctuation (ALFF) in the right inferior frontal cortex, left sensorimotor cortex, bilateral cerebellum, and the vermis, as well as increased ALFF in the right anterior cingulate cortex, left sensorimotor cortex, and bilateral brainstem.

While group level analysis can suggest statistical differences between two groups, it may not be that useful for clinical diagnosis at the individual level. There have been relatively few investigations at the individual level of classification of the ADHD subjects. One such study is performed by Zhu et al. (2008) who used a PCA-LDA (principal component analysis-linear discriminant analysis) based classifier to separate ADHD and control subjects at individual level. Unlike our network connectivity feature, which can connect all the synchronous regions of the whole brain, they used a regional homogeneity based feature for classification. Also the experiments were performed on only 20 subjects, which are not conclusive.

**Figure 1:**
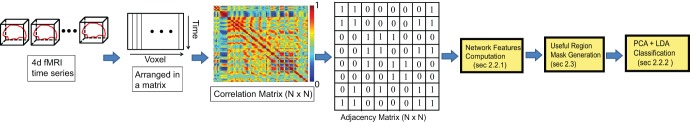
**Overview of our approach**. Compute an *N* × *N* correlation matrix (*N* is the number of voxels) using fMRI data; compute the adjacency matrix by thresholding the low correlation values to generate a network; compute network features such as node degree and cycle count for each node of the network; generate a mask for the brain regions which are believed to be most effective for classification; extract feature values within the generated brain mask and classify subjects using the PCA-LDA classifier.

Our algorithm exploits the topological differences between the functional networks of the ADHD and controlled brains. The different steps of our approach are described in the Figure 1. The input to our algorithm is brain fMRI sequences of the subjects. fMRI data can be viewed as a 4-D video such that the 3-D volume of the brain is divided into small voxels and imaged for a certain duration. The data can also be viewed as a time series of intensity values for each of the voxels. The correlation of these intensity time-series can be an indication of how synchronous the activities of two voxels are, and higher correlation values suggest that two voxels are working in synchronization. A functional network structure is generated for the brain of each of the subjects under study by computing the correlations for all possible pairs of voxels and establishing a connections between any pairs of voxels if their correlation value is sufficiently high. Different network features, such as degree maps, cycle maps, and weight maps are computed from the network to capture topological differences between ADHD and control subjects. We have provided a detailed description of all the network features in the later sections of the article. A brain mask is computed that includes only the regions with useful information to classify ADHD and control subjects. For the rest of the article, we refer to this mask as a “useful region mask.” The details of the useful region mask computation procedure are described in section 2.2. Finally, the network features from the voxels within the useful region mask are extracted to train a PCA-LDA based classifier. We have tested the performance of each of the network features computed on the training data set from the Kennedy Krieger Institute (KKI). We selected two different kinds of network features, degree map and 3-cycle map, for the experiments on the full data set.

In our work, we have performed experiments on a large challenging data set which includes subjects from different races, age groups, and data capturing sites. We propose a new approach for the automatic classification of ADHD subjects, and believe that our work will be helpful to the medical imaging community.

## 2. Materials and methods

### 2.1. Data

We use the data provided by the Neuro Bureau for the ADHD 200 competition which consists of 776 training subjects and 197 test subjects. Eight different centers contributed to the compilation of the whole data set, which makes the data diverse as well as complex. Different phenotypic information, such as age, gender, handedness, and IQ, is also provided for each subject. Consider Table 1 for an overview of the data set. All research conducted by ADHD-200 data contributing sites was conducted with local IRB approval, and contributed in compliance with local IRB protocols. In compliance with HIPAA Privacy Rules, all data used for the experiments of this article is fully anonymized. The competition organizers made sure that the 18 patient identifiers are removed, as well as face information.

**Table 1:** **Summary of the data set released for ADHD-200 competition**.

**Center**	**Sub Cnt**	**Age (years)**	**Male**	**Female**	**Control**	**Combined**	**Hyperactive**	**Inattentive**
**TRAINING DATA SET**								
Kennedy Krieger Institute	83	8 − 13	46	37	61	16	1	5
Neuro Image Sample	48	11 − 22	31	17	23	18	6	1
New York University	222	7 − 18	145	77	99	77	2	44
Oregon Health and Science University	79	7 − 12	43	36	42	23	2	12
Peking University	152	8 − 17	102	50	93	22	0	37
University of Pittsburg	89	10 − 20	46	43	89	0	0	0
Washington University in St. Louis	61	7 − 22	33	28	61	0	0	0
**TEST DATA SET**								
Kennedy Krieger Institute	11	8 − 12	10	1	8	3	0	0
Neuro Image Sample	25	13 − 26	12	13	14	11	0	0
New York University	41	7 − 17	28	13	12	22	0	7
Oregon Health and Science University	34	7 − 12	17	17	27	5	1	1
Peking University	51	8 − 15	32	19	27	9	1	14
University of Pittsburg	9	14 − 17	7	2	5	0	0	4
Brown University	26	8 − 18	9	17	−	−	−	−

A total of eight centers contributed to the data. The labels of the Brown University test set are not yet released.

For all our experiments we have used preprocessed resting state fMRI data registered in a 4 × 4 × 4 mm voxel resolution Montreal Neurological Institute (MNI) space, with nuisance variance removed, filtered using a bandpass filter (0.009Hz <f <0.08 Hz) and blurred with a 6-mm FWHM Gaussian filter. All the fMRI scans are motion corrected to the first image of the time series. We have used a binary mask, provided with each of the subjects, to find out the voxels inside the brain volume. All the fMRI data volumes are of size 49 × 58 × 47 voxels, but the number of sample across the time vary based on the center where data is captured. Further information regarding the data and the preprocessing steps is provided in NITRC (2011).

Though no quality control is performed on the data, a quality score is provided with each image file of all the subjects. The voxel-wise z-scores are thresholded and summed over all the voxels to compute the quality score of a image file. Images with low scores are considered to be better. We have not considered the quality scores for our study.

### 2.2. Method

Network motifs such as node degree distribution, cycle, etc. are analyzed in different disciplines of science to understand the systems being studied and neuroscience is not an exception (Milo et al., 2002; Ma'ayan et al., 2008; Sporns, 2002). We used different graph theoretic concepts for our study. We assume that the activity of a brain can be modeled as a functional network where the voxels are considered as the nodes, which are connected with each other based on the similarity of their activity over the time domain. In this article we have used the terms voxel and node interchangeably for the same meaning. The time series of a node is represented as a bold face notation. As the first step of the algorithm, we extract the time series for all the voxels and reorganized it as a separate 2-D matrix for each of the subjects in the data set. This is illustrated in second step of Figure 1. Next, the correlation between all possible voxel pairs is computed. If a subject contains *N* number of voxels, a correlation matrix of size *N* × *N* is constructed, where the *i*th row of the matrix corresponds to the pairwise correlation values of the *i*th voxel with all other voxels within the anatomical mask of the subject.

For any two voxels, if the time series are **u** and **v**, respectively, the correlation can be computed as,

(1) $$r=\frac{\left(T\sum_{i=1}^{T}u_{i}v_{i}\right)-\left(\sum_{i=1}^{T}u_{i}\right)\left(\sum_{i=1}^{T}v_{i}\right)}{\sqrt{\left[T\sum_{i=1}^{T}u_{i}^{2}-{\left(\sum_{i=1}^{T}u_{i}\right)}^{2}\right]\left[T\sum_{i=1}^{T}v_{i}^{2}-{\left(\sum_{i=1}^{T}v_{i}\right)}^{2}\right]}},$$

where *T* is the length of the time series, **u** = [*u*_1_, *u*_2_, …, *u*_*T*_], **v** = [*v*_1_, *v*_2_, …, *v*_*T*_].

We normalize all the time series between [−1, 1] before correlation computation. Next, we threshold all the values of the correlation matrix to get a binary map of zeros and ones. This binary map can be considered as the adjacency matrix of a graph where the *i*th voxel is connected to all the voxels for which non-zero values are present in the *i*th row of the matrix. Note that we can consider two voxels to be connected by an edge when the correlation is high positive, high negative or simply the absolute value of the correlation is high. We have computed three different networks considering high positive, high negative, and high absolute correlation values, respectively.

#### 2.2.1. Network feature computation

Once the graphs are constructed, for each subject of the data set, we compute different network features which can provide certain functional differences between the activity patterns of ADHD and control subjects' brain. The feature values from all the voxels of a network construct the feature map such as degree map, cycle map, etc. The descriptions of different network features computed are given below.

***2.2.1.1. Degree*** For each node in a network, the degree is the count of the other nodes it is connected to. In other words, the degree of a node is the number of edges attached to it.

**Figure 2:**
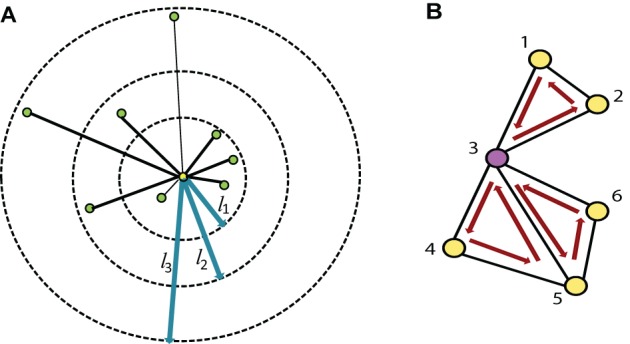
**(A)** The degree of the node, highlighted in yellow, is the count of all the green nodes connected to it (i.e., 8), while the varying distance degree is the counts of all the connected nodes in each of the bins defined by the three edge length thresholds (*l*_1_, *l*_2_, *l*_3_) marked in blue. In this example the varying distance degrees of the yellow node are {4, 2, 2}. **(B)** Shows all the distinct 3-cycles that containing the node 3.

***2.2.1.2. Varying distance degree*** Instead of considering the count of all the edges of a node as its degree, we group the edges based on their physical length and compute a separate degree for each of the groups. So, if we have *n* threshold values for edge length, say {*l*_1_, *l*_2_, …, *l*_*n*_}, we can compute *n* degrees, {*d*_1_, *d*_2_, …, *d*_*n*_}, of a node *v*, where *d*_*i*_ is the count of all the edges connected to *v* with length between *l*_*i* − 1_ to *l*_*i*_. Refer to the Figure 2 for details. We use the Euclidian distance measure for the calculation of edge length. For the experiments, we have used threshold values 20, 40, and 80 mm. where the average brain volume is approximately of size 172×140×140 mm. Hence, we get 4° per node which count edges of length 0–20, 20–40, 40–80, and greater than 88 mm, respectively. The thresholds are selected through an intuitive basis such that different degrees should capture local to global connectivity pattern. The average percentage of degrees from close to far range are found as 70.44%, 16.54%, 8.40%, and 4.62%.

***2.2.1.3. L-cycle count*** A path in a network is a sequence of distinct nodes which can be traversed in the given order using the connecting edges. A cycle, on the other hand, is a closed path in the network where the starting and ending node is the same and all other nodes are distinct. The L-cycle count of a node is the number of all possible distinct *L* length cycles containing the node. Figure 2 illustrates this idea. L-cycle count for a node is calculated by traversing through all the L-length path starting from the node and counting the paths which leads to the starting node. The traversing can be performed using the breadth first search algorithm. We have used different cycle lengths for our experiments.

***2.2.1.4. Weight sum*** Instead of constructing an adjacency matrix using a threshold on the correlation values, we assume every node is connected to all other nodes by the weighted edges. The weight of the connecting edge of a node pair is their correlation value. As the correlation values can be positive and negative, we can separately add up all the positive, negative and absolute edge weights of a node to get its sum of positive, negative and absolute weights.

#### 2.2.2. PCA-LDA classification

Once we finish computation of the network features, we extract the features from all of the voxels within the useful region mask. The mask generation algorithm is described in the next subsection. Concatenation of the feature values extracted from all the voxels generates a feature vector per subject. A PCA-LDA based classifier is trained separately using different set of feature vectors computed for different types of network features. Finally, the classifier is used for automatic classification of the ADHD subjects.

It is expected that the characteristics of the networks computed are represented by their feature vectors. A feature vector of a network represents a point in the feature space where the dimensionality of the space is same as the length of the vector. If the feature vectors of ADHD and control subjects are separable then their corresponding points in the feature space should cluster in different locations. When a classifier is trained, it learns to partition the feature space in such a way that the feature vectors from each of the groups are ideally clustered in separate segments. Given a feature vector of a test example, the classifier can identify which segments of the feature space it belongs to and classify the test subject accordingly. LDA is a widely used data classification technique which maximizes the ratio of between-class variance to the within-class variance to produce maximal separability. Mathematically, the objective is to maximize the following function:

(2) $$J(w)=\frac{w^{T}S_{B}w}{w^{T}S_{W}w}$$

where *S*_*B*_ and *S*_*W*_ are between class and within class scatter matrix, and can be formulated as follows:

(3) $$S_{B}=\sum_{i=1}^{n_{A}}{\left(x_{i}{}^{\left(A\right)}-\mu^{\left(A\right)})(x_{i}{}^{\left(A\right)}-\mu^{\left(A\right)}\right)}^{T}+\sum_{i=1}^{n_{C}}{\left(x_{i}{}^{\left(C\right)}-\mu^{\left(C\right)})(x_{i}{}^{\left(C\right)}-\mu^{\left(C\right)}\right)}^{T}$$

(4) $$S_{W}=\left(\mu^{\left(A\right)}-\mu^{\left(C\right)}\right){\left(\mu^{\left(A\right)}-\mu^{\left(C\right)}\right)}^{T},$$

*n*_*A*_ and *n*_*C*_ are the number of subjects, μ^(*A*)^ and μ^(*C*)^ are the mean feature vectors, *x*^*A*^_*i*_ and *x*^*C*^_*i*_ are the *i*th feature vectors of the ADHD and control group, respectively.

In many cases, the dimension of the feature space becomes so high that the proper partitioning of the space is difficult. For example, in our case, the dimension of the feature space is the number of voxels within the useful region mask which is several thousands. Again, most of the dimensions do not contain any significant data variance. PCA is a procedure to find out a set of orthogonal directions, called principal components, along which the variance of the data is maximum. It then projects the data into the smaller dimensional subspace composed of the principal components. The classifier can work efficiently on the subspace which is significantly smaller in dimension than the original feature space. We use first 40 and first 100 principal components for the experiments on KKI and full data set, respectively, as they cover more than 98% of data variance. We have included a plot of principal component vs. percent of data variance in the supplementary materials. Refer to Abdi and Williams (2010) for details about PCA.

### 2.3. Useful region mask

Different research studies have proposed several regions of interests (ROI) for fMRI analysis. These different ROIs vary in size and number. In some studies they are identified based on the anatomical structure of the brain and in other studies they depend on the functional responsibility. Tzourio-Mazoyer et al. (2002) identified the ROIs based on similar functional responses in the brain. Craddock et al. (2011) generated a homogenous functional connectivity map from resting state fMRI data. Smith et al. (2009) identified several co-varying functional subnetworks in the resting state brain. However, it is still unclear which ROIs are the best for resting state functional connectivity analysis. Also it is not known if all the ROIs detected by one method are required for ADHD classification or if the use of a subset of ROIs is more efficient. To find these answers, we use a novel method to identify the useful region mask for the classification of ADHD and control subjects. The algorithm for the useful region mask generation is as follows:

**Step 1** For each of the subjects, used for mask generation algorithm, we do the following:
  - Divide the brain volume into small cube-shaped regions. Each of the regions is typically 5 × 5 × 5 voxels except the regions at the boundary of the brain volume.
  - Select a random subset of the regions. We include each region in the subset with probability *p*.
  - Generate degree map by extracting the degrees for the voxels within the selected subset of regions.
**Step 2** Train the PCA-LDA based classifier and calculate the detection accuracy on the test data set.
**Step 3** Perform the step 1 and step 2 for *m* number of times, each time generating a different random subset, calculating the detection accuracy and recording it.
**Step 4** Choose the random sub sets corresponding to the top 10% of the detection accuracy as the candidates for generating the useful region mask. We count the occurrence of each of the regions in all of the candidate sub sets and normalize the counts between 0 and 1 by dividing it by the number of candidate sub sets. This gives us the probability of inclusion of each of the regions in the mask.
**Step 5** Finally the useful region mask is generated using a threshold *th* to prune the regions with low probability.

**Figure 3:**
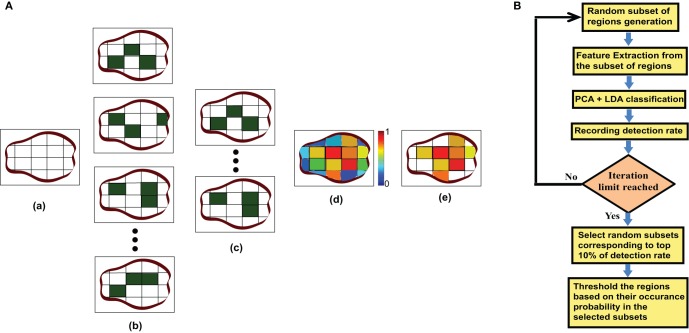
**(A)** This part of the figure explains the useful region mask generation algorithm on a single brain slice. The figure is just a graphical example, not the real data. In actual experiments brain volumes are used instead of slices and cube regions are used instead of square subdivision areas. (a) Divide the slice into square regions. (b) Select random sub sets of square regions marked in dark green. (c) Select the sub sets with top 10% of detection rate. (d) Generate a probability map based on the regions occurrence in top 10% subset. (e) Threshold the probability map to produce the useful region mask. **(B)** This part shows the flowchart for the mask generation algorithm.

We experimentally verified that highest detection rate achieved when *p* is 0.40 and *th* is 0.60. The experiment results are included in the supplementary materials. The value of *m* was kept as 500 so that the number of iterations should be large enough but computationally feasible. Figure 3A is an illustration of the proposed algorithm on a cartoon 2-D slice of a brain while Figure 3B is the flowchart for the mask generation algorithm. Note that other network features may also be used in the algorithm but we simply use degree map feature. We assume that the regions, which are useful for identifying ADHD conditioned brains, should not vary depending on the feature used for the detection of the mask. We have tested the idea computing useful region mask using 3-cycle map feature also. We found that the final detection rates are very similar (check the supplementary materials).

## 3. Experiments and results

**Figure 4:**
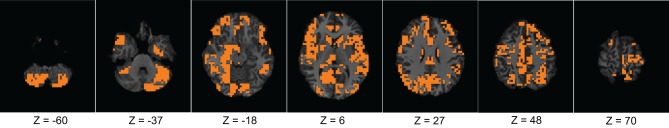
**The figure shows different slices to demonstrate the useful region mask computed**. The masked regions are highlighted in orange color and overlaid on the structural images of a sample subject.

**Figure 5:**
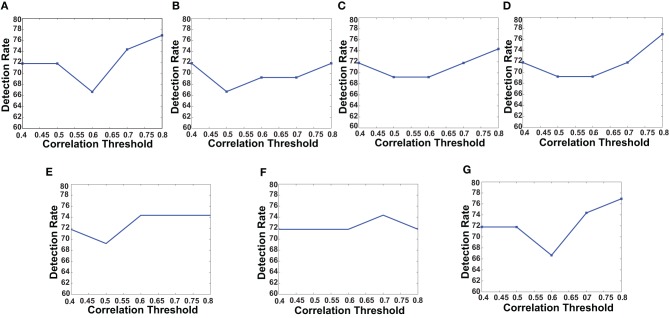
**The plots shows how detection rates for different network features change with correlation threshold**. **(A)** Degree map positive correlations, **(B)** degree map negative correlations, **(C)** degree map absolute correlations, **(D)** varying distance degree map positive correlation, **(E)** 3-cycle map positive correlation, **(F)** 4-cycle map positive correlation, and **(G)** weight map positive correlation.

First, we verified the performance of each of the network features computed on a subset of the training data. We used fMRI data of 83 subjects from the KKI data set. Among the 83 subjects, the first 44 subjects are used for training and the remaining 39 for testing. The performances of each of the features is computed with or without using the useful region mask. The mask is generated on the KKI training set comprising the first 44 subjects of the KKI subset and using the algorithm described in section 2.3. Each time a random subset of regions is selected, the classification performance is measured by leave-one-out cross verification, i.e., take 43 subjects for training and test on the remaining one subject, repeat the process 44 times, testing each of the 44 subjects one at a time and averaging the correct detection count. Figure 4 shows the computed mask on different slices of the brain. Table 2 list the information of the different clusters found in the useful region mask and the ROIs they are overlapped with. To empirically select the correlation threshold to be used for our experiments, we varied it from 0.4 to 0.8 with an increment of 0.1 in every step. In each step, detection rates for different network features are computed on the KKI test set of 39 subjects. The plots for correlation threshold vs. detection rate are shown in Figure 5. To generate the plot for the weight map, we compute the sum of the edge weights considering only the edges which have weights greater than the correlation thresholds used within that step. Note that the detection rate for each feature is measured for positive, negative and absolute correlation values. However, the features computed from the positive correlation values have always outperformed the other two cases. Hence, we have not reported the other two cases in the paper. Since for all the network features, other than the 4-cycle map, the best performance is consistently achieved when correlation threshold is 0.80, we choose to use this value for all the experiments on the full data set.

**Table 2:** **Shows list of the clusters and their approximate centers, sizes and standard deviations found using the most useful region mask algorithm**.

**ROIs**	**[x, y, z] centers in mm**	**Size in mm^3^**	**Standard deviation in mm**		
			**x**	**y**	**z**
Precuneus cortex	[0, −66, 42]	7872	5.4894	6.6435	10.3592
Cingulate gyrus	[0, −36, 52]; [0, 6, 42]	13,056	4.5593	11.3751	10.9128
Temporal pole	[56, 14, −18]	5312	4.7728	5.5878	5.7664
Superior temporal gyrus	[60, −18, −8]; [−60, −20, −4]	3392; 6400	7.1938; 6.6817	9.4413; 11.6393	4.0790; 5.7075
Inferior temporal gyrus	[54, −30, −20]; [−60, −48, −10]	1856; 2816	7.6293; 5.4892	6.7262; 8.2390	8.2617; 5.3582
Pre-central gyrus	[−6, −22, 62]	8000	16.7226	8.5099	5.2886
Lingual gyrus	[6, −64, 4]	19,072	12.5240	11.4946	5.8835
Right amygdala	[24, −2, −18]	2176	9.6639	7.3186	7.1020

The coordinates are calculated on the HarvardOxford-cort-maxprob-thr0-1 mm standard atlas provided with the FMRIB Software Library (FSL) 4.1. We list the ROIs of Harvard–Oxford cortical and subcortical structural Atlases for which more than 50% of the volumes are selected in the useful region mask. Atlas tool of FSL view is used for this purpose.

Table 3 summarizes the best performance obtained for each of the network features and the corresponding correlation threshold values. The performance in the table signifies the percentage of total number of correct detection (control and ADHD) among total number of test subjects. Note that for all the features, the performance without using useful regions mask is lower compare to when we use the mask. This demonstrate the utility of the voxel selection through the generated mask. In one of the recent studies Solmaz et al. used Bag of Word features for automatic classification of the ADHD subjects (Solmaz et al., 2012). We used their method for the purpose of comparison of the performances with our method. For our experiments using the Bag of Words feature, each subject is represented by 75 and 100 bin histograms when we used raw time series and degree map features, respectively. A third kind of experiment performed by representing each of the subjects as a concatenation of two types of histograms resulting in a 175 bin histogram. The details of the Bag of Word method are provided in the supplementary materials.

**Table 3:** **Initial test results shows the performance of all the network features computed on the Kennedy Krieger Institute's data set**.

**Feature**	**Correlation threshold**	**Performance (%) using useful region mask**	**Performance (%) without useful region mask**
Degree map positive	0.80	76.92	69.23
Degree map negative	0.80	71.79	69.23
Degree map absolute	0.80	74.36	71.79
Varying distance degree map	0.80	76.92	69.23
3-cycle-map	0.80	74.36	71.79
4-cycle-map	0.70	74.36	69.23
Weight map positive	0.80	76.92	69.23
BOW time series histogram	-	69.23	66.67
BOW degree map histogram	0.80	69.23	66.67
BOW time series and degree map histogram	0.80	69.23	66.67

Positive, negative and absolute keywords are used to indicate that positive, negative and absolute correlation values are considered for network generation. If any keyword is not specifically mentioned, then the positive correlation values are used only.

We perform thorough experiments on the full data set using positive degree map and positive 3-cycle map features. We trained our classifier with the full training data, which has 776 subjects from 7 different centers, and test on the 171 subjects from 6 centers released for the ADHD-200 competition. Again, we compared the performance with and without using the useful region mask. We reused the same mask generated using first 44 subjects of KKI. It is worth mentioning that the mask selects 6916 voxels from which features are extracted. The correct detection rate, specificity and sensitivity for each of the test centers and for overall centers are reported in Table 4. Since the subject labels of the Brown University test set have not yet been released, we cannot compute the performance measures on that subset.

**Table 4:** **Shows the detection rate, specificity and sensitivity of the classification experiments on the test data set released for the ADHD-200 competition**.

	**Degree map (mask)**			**Degree map (no mask)**			**3-cycle map (mask)**			**3-cycle map (no mask)**		
	**Detection rate**	**Specificity**	**Sensitivity**	**Detection rate**	**Specificity**	**Sensitivity**	**Detection rate**	**Specificity**	**Sensitivity**	**Detection rate**	**Specificity**	**Sensitivity**
KKI	72.72	1	0	72.72	1	0	72.72	1	0	72.72	1	0
Neuro image	68	0.7857	0.5454	64	0.7143	0.5454	72	0.7857	0.6364	68	0.8572	0.4545
NYU	70.73	0.9167	0.6207	65.85	0.7500	0.6207	70.73	0.8333	0.6552	63.41	0.8333	0.5517
OHSU	70.59	0.7778	0.4286	64.70	0.7037	0.4286	73.52	0.8148	0.4286	70.59	0.7407	0.5714
Peking	64.71	0.8889	0.3750	60.78	0.8889	0.2917	62.74	0.9259	0.2917	56.86	0.9630	0.1250
Pittsburgh	77.78	1	0.5000	66.67	0.8000	0.5000	77.78	1	0.5000	66.67	1	0.2500
Overall	69.05	0.8602	0.4872	64.32	0.7957	0.4615	69.59	0.8710	0.4872	64.33	0.8710	0.3718

Comparison of the performances are shown when useful region mask is used and not used for the degree map and 3-cycle map features.

## 4. Discussion

**Figure 6:**
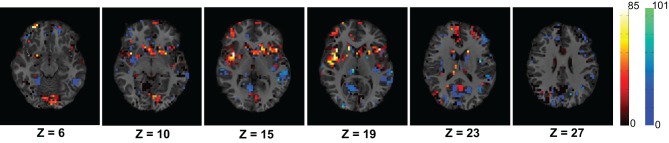
**The figure shows average difference of degrees of the control group from the ADHD group for the voxels within the useful region mask**. The average difference is calculated using the 83 subjects of KKI training set. The dark red to white color map is used to represents higher degree of control subjects and blue to green color map is used to show the opposite. The control group shows higher connectivity in the Cingulate Gyrus region on slices with Z coordinates 10 and 15 and Paracingulate Gyrus region on slices with Z coordinates 19 and 23.

We have modeled the brain as a functional network which is expected to represent the interaction of the different active regions of the brain. We assumed that ADHD is a problem caused due to the partial failure of the brain's communication network and the affected subjects can be distinguished from control subjects using the topological differences of their respective functional networks. To verify the idea, we have extracted different network features to train a PCA-LDA based automatic classifier. Figure 6 shows that the average degree map, computed for the ADHD and control subjects of the KKI data set, is able to capture some difference of connectivity in the Cingulate Gyrus and the Paracingulate Gyrus regions of brain. We also proposed that the features from the whole brain are not required for the classification, but some key areas hold useful information. Our results shows that the inclusion of features from the whole brain can negatively impact the classification accuracy. This resulted in a novel algorithm to compute the useful region mask which helped to improve the classification performance.

The different network features computed are expected to capture different characteristics of the functional network. The degree map and the weight map can capture how densely the nodes of the network is connected. This can give us a measure of how synchronously different regions of a brain are interacting. Varying distance degree map, on the other hand, can also reveal the fact that how the synchronous regions are distributed over the brain. While degree map only captures pairwise interactions of voxels, it ignores higher-order interactions, such as among three voxels simultaneously. We know from brain anatomy that there are such multiply connected brain regions. Hence, cycle maps offer a different perspective from which a given network may be viewed. The utility of using network motifs such as cycles to describe networks has been described in Milo et al. (2002).

Figure 4 and Table 2 presents the ROIs found through our adaptive labeling technique described in section 2.3. These ROIs were used in the classification including regions such as the cingulate and precuneus which is consistent with the findings of Castellanos et al. (2008). The cingulate and precuneus regions are known to be part of the DMN (Damoiseaux et al., 2006). Many regions in the Table 2 have also been identified by Assaf et al. (2010), such as the precuneus, temporal pole, superior temporal gyrus, and pre-central gyrus. Regions in Table 2 that are consistent with those reported by Uddin et al. (2009) include the inferior temporal gyrus and lingual gyrus. Interestingly, Table 2 identifies the right amygdala, which did not show up in the analysis of Castellanos et al. (2008) or Assaf et al. (2010) or Uddin et al. (2009). The limbic system is known to play a role in ADHD, and a study by Plessen et al. (2006) reported disrupted connectivity between the amygdala and OFC in the children with ADHD. Hence the value of our technique is that it provides an independent and automatic source of hypotheses about the brain regions that are implicated in the diagnosis and classification of ADHD. In this sense, our technique for ROI identification can be considered to be a model-free method. Furthermore, our classifier is agnostic to any particular theory of ADHD, and works strictly on a machine-learning approach to separating ADHD patients from controls by utilizing labeled data. Hence the technique described in this paper is applicable to other types of brain disorders where one can create labeled data for the accompanying brain scans.

The curves in Figure 5 show that for all the network features, high performance value is achieved when correlation threshold 0.80 is used to construct the network. In four out of seven cases the performances are the highest, in other two cases they are one of the highest and in one case it is slightly lower that the highest. The results are not surprising since they indicate that the difference of connection structure for highly correlated voxels matters the most for classification.

Considering the results in Table 4, we observe that in five out of six data sets, the 3-cycle maps with voxel selection give the best detection rate. Only on one data set, the Peking data set, the 3-cycle map with voxel selection gives marginally worse performance than the degree map with voxel selection. To the best of our knowledge, this is the first time that the utility of cycle-related features has been demonstrated in the fMRI imaging literature. The study in Ma'ayan et al. (2008) showed that cycle-related features are useful in discriminating biological networks from man-made networks, but did not investigate various types of fMRI-derived networks.

We note that calculating cycle-related features is more computationally intensive than the degree map, and the computation increases exponentially with cycle length. The use of GPUs can reduce the cost of computation, as earlier studies with fMRI images have shown Rao et al. (2011). If standardized libraries for cycle computation become available on GPU platforms, it will promote the use of such features in fMRI research.

The use of the degree map provides a good compromise between classification performance and computational cost. It is easy to compute, and provides classification performance that is only marginally worse than that of the 3-cycle maps in most cases. One limitation of our study is that we have not used any specific measure to remove different signal to noise ratios which may be introduced in the data due to the difference of experimental setups among the sites. Also, some of the recent studies (Power et al., 2012; Van Dijk et al., 2012) indicate that the correlations of different brain regions are sensitive to the motion of the head even though the data is preprocessed for motion correction. We have not performed any explicit step to counter this problem. Finally, we note that we used a single classifier, the PCA-LDA method to investigate the utility of different network features. It is possible that other classifiers such as neural networks or support vector machines may give better performance. Such investigations need to be carried out in the future.

### Conflict of interest statement

The authors declare that the research was conducted in the absence of any commercial or financial relationships that could be construed as a potential conflict of interest.

## Figures and Tables

**Figure A1:**
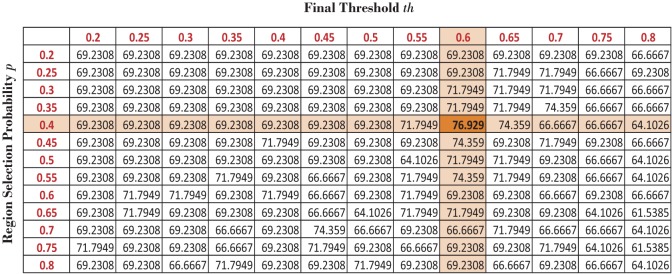
**Different detection results on KKI data set based on different set of values of *p* and *th***.

**Figure A2:**
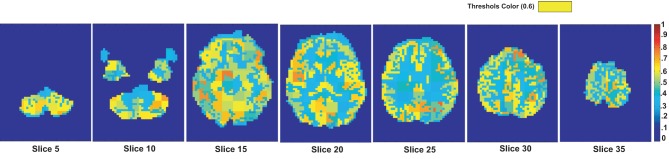
**The region occurrence probability map generated during the useful region mask computation on the KKI data set using positive degree map features**.

**Figure A3:**
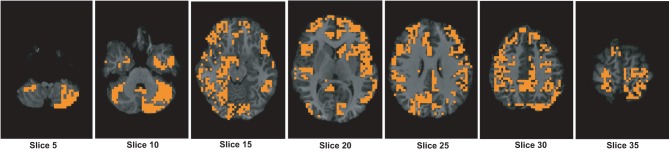
**The figure shows different slices to demonstrate the useful region mask computed using 3-cycle map features**. The masked regions are highlighted in orange color and overlaid on the structural images of a sample subject.

**Figure A4:**
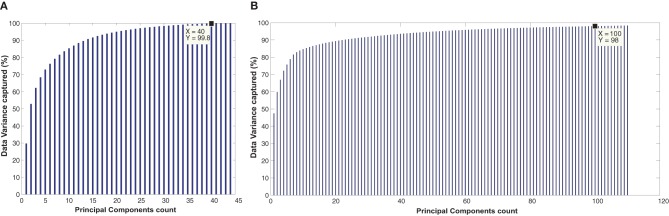
**The figure shows the plots of principal component count vs. percentage of data variance for (A) KKI training data (B) full training data of 776 subjects**.

**Figure A5:**
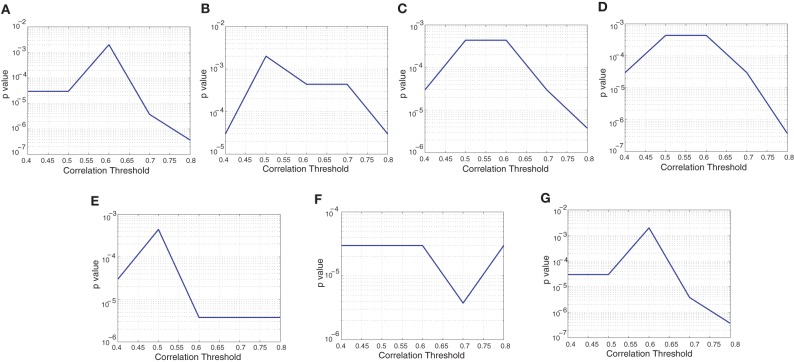
**The plots shows *p*-values corresponding to the detection rates for different network features and different correlation thresholds. (A)** Degree map positive correlations, **(B)** degree map negative correlations, **(C)** degree map absolute correlations, **(D)** varying distance degree map positive correlation, **(E)** 3-cycle map positive correlation, **(F)** 4-cycle map positive correlation, and **(G)** weight map positive correlation.

## Appendix

### Experiments for finding best *p* and *th* value

We varied the probability *p* of including a region in the random subset and the final threshold *th* used on the occurrence probability map of the regions to generate different useful region mask. Please check the useful region mask generation algorithm in the section 2.3 of the main article for the details of *p* and *th*. For each pair of values of *p* and *th*, we compute a different useful region mask which is used to generate different detection rates on the KKI data set. The detection rates are reported in the Figure A1. The best performance is achieved when *p* = 0.4 and *th* = 0.6. We used these values for the generating the final useful region mask.

### The map of region occurrence probability

In the useful region mask computation algorithm in the section 2.3 of the main article we used top 10% of the random subsets generated as the candidate subsets for generating the final mask. We compute the number of occurrence of each of the regions in the candidates subsets and divide it by the total number of candidate subsets to generate the region occurrence probability map. This map is reported in the Figure A2 as per the request of reviewer 2.

### Useful region mask using 3-cycle map feature

We assume that the regions, which are useful for identifying ADHD conditioned brains, should not vary depending on the feature used for the detection of the mask. To justify our assumption we generate another useful region mask on the KKI data set using 3-cycle map features. The mask generated is used to verify the detection rates on the test data sets released for the ADHD-200 competition. The experiment results are reported in the Table A1. The detection rates we got using the mask generated with 3-cycle map features and using the mask generated with positive degree map features are almost same. This matching results supports our initial assumption. The Figure A3 shows the mask plotted on the different slices of a brain.

**Table A1:** **Shows the detection rate of the classification experiments on the test data set released for the ADHD-200 competition**.

	**Detection rate (%)**	**Specificity**	**Sensitivity**
KKI	72.72	1	0
Neuro Image	72	57.14	90.91
NYU	70.73	0.8333	0.6552
OHSU	73.52	0.8889	0.1429
Peking	60.78	0.9630	0.1667
Pittsburg	77.78	1	0.5000

The useful region mask, generated using 3-cycle map features, is used for identifying the regions to extract the features.

### Principal component and data variance analysis

The Figure A4 show the plots for the number of principal components vs. the percentage of the total data variance captured. For the KKI training data set, the first 40 principal components are able to capture 99.8% of total data variance while the first 100 principal components of the full training data set are able to capture 98% of the total data variance.

### Bag of words for ADHD classification

Bag of Word (BoW) model was first introduced in natural language processing. The main idea of BoW is that a document can be represented by the histogram of the counts of different words consisting the document. The order of the words or the grammar of the language is immaterial. Again, different documents on the same topic should share similar histogram pattern while the patterns of the histograms of the documents on different topics should differ.

A similar idea is used by Solmaz et al. (2012) for the classification of ADHD subjects. For the purpose of constructing vocabulary of the resting state fMRI data, the authors cluster the time series of the voxels in all the subjects of the training set. This step groups the similar time series in the same group. The mean time series for each of the groups construct the vocabulary of the fMRI data. Hence, we can say that the number of clusters formed is the number of different words the resting state fMRI data can have. Now given anytime series of a voxel it can be labeled to the group number of the closest group. Hence, each subject can be represented as a histogram of word count based on how many voxels of the subject belongs to which group. The histogram of the subjects serves as their feature vector. Now, given a training and test data set, a classifier can be trained on the histogram of the training subjects and used to classify the test subjects.

### *p*-values

The Figure A5 shows the *p*-values corresponding to the detection results shown in the Figure A4 of the paper. The *p*-value of a classification can be interpreted as the probability that the classification accuracy can be achieved if the classifier is random. For example, if *m* subjects are correctly classified among *n* test subjects then *p*-value for the classification would be the probability of *m* or more correct detections if classifier detects using random chance. The lower the *p*-value the lower the chance that the classification is not random.

## References

[B1] Abdi H. Williams L. J. (2010). Principal component analysis. Wiley Interdiscip. Rev. Comput. Stat. 2, 433–459.

[B2] Assaf M. Jagannathan K. Calhoun V. D. Miller L. Stevens M. C. Sahl R.. (2010). Abnormal functional connectivity of default mode sub-networks inautism spectrum disorder patients. Neuroimage 53, 247–256. 10.1016/j.neuroimage.2010.05.067 20621638 PMC3058935

[B3] Bush G. Frazier J. A. Rauch S. L. Seidman L. J. Whalen P. J. Jenike M. A.. (1999). Anterior cingulate cortex dysfunction inattention-deficit/hyperactivity dis-order revealed by fMRI and the countingstroop. Biol. Psychiatry 45, 1542–1552. 10376114 10.1016/s0006-3223(99)00083-9

[B4] Cao Q. Zang Y. Sun L. Sui M. Long X. Zou Q.. (2006). Abnormal neural activity in children with attention deficithyperactivity disorder: a resting-state functional magnetic resonance imagingstudy. Neuroreport 17, 1033–1036. 10.1097/01.wnr.0000224769.92454.5d 16791098

[B5] Castellanos F. X. Giedd J. N. Marsh W. L. Hamburger S. D. Vaituzis A. C. Dickstein D. P.. (1996). Quantitative brain magnetic resonance imaging in attention-deficithyperactivity disorder. Arch. Gen. Psychiatry 53, 607–616. 10.1001/archpsyc.1996.01830070053009 8660127

[B6] Castellanos F. X. Margulies D. S. Kelly C. Uddin L. Q. Ghaffari M. Kirsch A.. (2008). Cingulate-precuneus interactions: a new locus of dysfunction in adultattention-deficit/hyperactivity disorder. Biol. Psychiatry 63, 332–337. 10.1016/j.biopsych.2007.06.025 17888409 PMC2745053

[B7] Cherkassky V. L. Kana R. K. Keller T. A. Just M. A. (2006). Functional connectivity in a baseline resting-state network inautism. Neuroreport 17, 1687–1690. 10.1097/01.wnr.0000239956.45448.4c 17047454

[B8] Craddock R. C. James G. Holtzheimer P. E. Hu X. P. Mayberg H. S. (2011). A whole brain fMRI atlas generated via spatially constrained spectralclustering. Hum. Brain Mapp. 33, 1914–1928. 10.1002/hbm.21333 21769991 PMC3838923

[B9] Damoiseaux J. S. Rombouts S. A. R. B. Barkhof F. Scheltens P. Stam C. J. Smith S. M.. (2006). Consistent resting-state networks across healthy subjects. Proc. Natl. Acad. Sci. U.S.A. 103, 13848–13853. 10.1073/pnas.0601417103 16945915 PMC1564249

[B10] Durston S. (2003). Differential patterns of striatal activation in young children withand without ADHD. Biol. Psychiatry 53, 871–878. 10.1016/S0006-3223(02)01904-2 12742674

[B11] Greicius M. D. Srivastava G. Reiss A. L. Menon V. (2004). Default-mode network activity distinguishes alzheimer's disease fromhealthy aging: evidence from functional MRI. Proc. Natl. Acad. Sci. U.S.A. 101, 4637–4642. 10.1073/pnas.0308627101 15070770 PMC384799

[B12] Ma'ayan A. Cecchi G. A. Wagner J. Rao A. R. Iyengar R. Stolovitzky G. (2008). Ordered cyclic motifs contribute to dynamic stability in biologicaland engineered networks. Proc. Natl. Acad. Sci. U.S.A. 105, 19235–19240. 10.1073/pnas.0805344105 19033453 PMC2614745

[B13] Milo R. Shen-Orr S. Itzkovitz S. Kashtan N. Chklovskii D. Alon U. (2002). Network motifs: simple building blocks of complex networks. Science 298, 824–827. 10.1126/science.298.5594.824 12399590

[B14] NITRC. (2011). Adhd-200 data processing. Available online at: http://nitrc.org/plugins/mwiki/index.php/neurobureau:Athena

[B15] Overmeyer S. Bullmore E. T. Suckling J. Simmons A. Williams S. C. Santosh P. J.. (2001). Distributed grey and white matter deficits in hyperkinetic disorder:MRI evidence for anatomical abnormality in an attentional network. Psychol. Med. 31, 1425–1435. 11722157 10.1017/s0033291701004706

[B16] Plessen K. J. Bansal R. Zhu H. Whiteman R. Amat J. Quackenbush G. A.. (2006). Hippocampus and amygdala morphology inattention-deficit/hyperactivity disorder. *Arch. Gen. Psychiatry* 63, 795–807. 10.1001/archpsyc.63.7.795 16818869 PMC2367150

[B17] Power J. D. Barnes K. A. Snyder A. Z. Schlaggar B. L. Petersen S. E. (2012). Spurious but systematic correlations in functional connectivity MRInetworks arise from subject motion. Neuroimage 59, 2142–2154. 10.1016/j.neuroimage.2011.10.018 22019881 PMC3254728

[B18] Raichle M. E. (2010). The brain's dark energy. Sci. Am. 302, 44–49. 20184182 10.1038/scientificamerican0310-44

[B19] Raichle M. E. MacLeod A. M. Snyder A. Z. Powers W. J. Gusnard D. A. Shulman G. L. (2001). A default mode of brain function. Proc. Natl. Acad. Sci. U.S.A. 98, 676–682. 10.1073/pnas.98.2.676 11209064 PMC14647

[B20] Rao A. R. Bordawekar R. Cecchi G. (2011). Fast computation of functional networks from fMRI activity: amulti-platform comparison. Proc. SPIE 7962, 79624L.

[B21] Seidman L. J. Valera E. M. Makris N. Monuteaux M. C. Boriel D. L. Kelkar K.. (2006). Dorsolateral prefrontal and anterior cingulate cortex volumetricabnormalities in adults with attention-deficit/hyperactivity dis-orderidentified by magnetic resonance imaging. Biol. Psychiatry 60, 1071–1080. 10.1016/j.biopsych.2006.04.031 16876137

[B22] Smith S. M. Fox P. T. Miller K. L. Glahn D. C. Fox P. M. MacKay C. E.. (2009). Correspondence of the brain's functional architecture duringactivation and rest. Proc. Natl. Acad. Sci. U.S.A. 106, 13040–13045. 10.1073/pnas.0905267106 19620724 PMC2722273

[B23] Solmaz B. Dey S. Rao A. R. Shah M. (2012). ADHD classification using bag of words approach on network features. Proc. SPIE 8314, 83144T.

[B24] Sowell E. R. Thompson P. M. Welcome S. E. Henkenius A. L. Toga A. W. Peterson B. S. (2003). Cortical abnormalities in children and adolescents withattention-deficit hyperactivity disorder. Lance 362, 1699–1707. 10.1016/S0140-6736(03)14842-8 14643117

[B25] Sporns O. (2002). Graph theory methods for the analysis of neural connectivity patterns, in Neuroscience Databases. A Practical Guide, ed Kötter R. (Boston, MA: Kluwer), 171–186.

[B26] Teicher M. H. Anderson C. M. Polcari A. Glod C. A. Maas L. C. Renshaw P. F. (2000). Functional deficits in basal ganglia of children withattention-deficit/hyperactivity disorder shown with functional magneticresonance imaging relaxometry. Nat. Med. 6, 470–473. 10.1038/74737 10742158

[B27] Tian L. Jiang T. Wang Y. Zang Y. He Y. Liang M.. (2006). Altered resting-state functional connectivity patterns of anteriorcingulate cortex in adolescents with attention deficit hyperactivitydisorder. Neurosci. Lett. 400, 39–43. 10.1016/j.neulet.2006.02.022 16510242

[B28] Tzourio-Mazoyer N. Landeau B. Papathanassiou D. Crivello F. Etard O. Delcroix N.. (2002). Automated anatomical labeling of activations in SPM using amacroscopic anatomical parcellation of the MNI MRI single-subject brain. Neuroimage 15, 273–289. 10.1006/nimg.2001.0978 11771995

[B29] Uddin L. Q. Clare Kelly A. Biswal B. B. Xavier Castellanos F. Milham M. P. (2009). Functional connectivity of default mode network components:correlation, anticorrelation, and causality. Hum. Brain Mapp. 30, 625–637. 10.1002/hbm.20531 18219617 PMC3654104

[B30] Van Dijk K. R. A. Sabuncu M. R. Buckner R. L. (2012). The influence of head motion on intrinsic functional connectivityMRI. Neuroimage 59, 431–438. 10.1016/j.neuroimage.2011.07.044 21810475 PMC3683830

[B31] Weissman D. H. Roberts K. C. Visscher K. M. Woldorff M. G. (2006). The neural bases of momentary lapses in attention. Nat. Neurosci. 9, 971–978. 10.1038/nn1727 16767087

[B32] Zang Y.-F. He Y. Zhu C.-Z. Cao Q.-J. Sui M.-Q. Liang M.. (2007). Altered baseline brain activity in children with ADHD revealed byresting-state functional MRI. Brain Dev. 29, 83–91. 10.1016/j.braindev.2006.07.002 16919409

[B33] Zhu C.-Z. Zang Y.-F. Cao Q.-J. Yan C.-G. He Y. Jiang T.-Z.. (2008). Fisher discriminative analysis of resting-state brain function forattention-deficit/hyperactivity disorder. Neuroimage 40, 110–120. 10.1016/j.neuroimage.2007.11.029 18191584

